# *Leishmania infantum* infecting the carnivore *Nasua nasua* from urban forest fragments in an endemic area of visceral leishmaniasis in Brazilian Midwest

**DOI:** 10.3389/fcimb.2022.1050339

**Published:** 2023-01-13

**Authors:** Gabriel Carvalho de Macedo, Wanessa Teixeira Gomes Barreto, Carina Elisei de Oliveira, Filipe Martins Santos, Grasiela Edith de Oliveira Porfírio, Samanta Cristina das Chagas Xavier, Fernanda Moreira Alves, Alanderson Rodrigues da Silva, Gisele Braziliano de Andrade, Andreza Castro Rucco, William Oliveira de Assis, Ana Maria Jansen, André Luiz Rodrigues Roque, Heitor Miraglia Herrera

**Affiliations:** ^1^ Post-Graduate Program in Environmental Sciences and Agricultural Sustainability, Dom Bosco Catholic University, Campo Grande, Brazil; ^2^ Post-Graduate Program in Biotechnology, Dom Bosco Catholic University, Campo Grande, Brazil; ^3^ Laboratory of Trypanosomatid Biology, Oswaldo Cruz Institute, Oswaldo Cruz Foundation, Rio de Janeiro, Brazil; ^4^ Post-Graduate Program in Parasite Biology, Oswaldo Cruz Institute, Oswaldo Cruz Foundation, Rio de Janeiro, Brazil

**Keywords:** longitudinal study, South American coati, *Leishmania infantum*, urban fauna, visceral leishmaniasis

## Abstract

**Introduction:**

The aim of the present study was to investigate the occurrence of *Leishmania infantum* in South American coatis inhabiting two forest fragments in Campo Grande, Mato Grosso do Sul, Midwest region of Brazil, an endemic area of human and canine visceral leishmaniasis (VL).

**Material and methods:**

A total of 110 South American coatis were sampled in the conservation unit “*Parque Estadual do Prosa*” (PEP) and in the residential area “*Vila da Base Aérea*” (VBA) from March 2018 to April 2019. As a longitudinal study that include up to six recaptures of the same individual, a total of 190 capture events were obtained. Blood, bone marrow and skin samples were obtained for parasitological (axenic culture), serological (Enzyme Linked Immunosorbent Assay – ELISA and Dual-path Platform immunoassay – DPP^®^ CVL) and molecular diagnostic assays (targeting kDNA for *Leishmania* spp. and *L. infantum*; and HSP70 followed by sequence analysis).

**Results:**

Seropositivity for *L. infantum* was found in 33 individuals, six in PEP and 27 in VBA. Furthermore, *L. infantum* was detected by molecular analysis in 16 individuals, seven from PEP and nine from VBA. We also isolated *L. infantum* from bone marrow of one individual and detected a single positive skin sample in molecular assay from other individual, both from VBA.

**Discussion:**

An overall infection rate of 36.4% (40/110) was observed, significantly higher in the VBA (49.1%) than in the PEP (21.6%), probably because VBA presents: (i) a large number of resident dogs and chickens that would be attracting sandflies; (ii) a denser population of this wild mammal species; and (iii) physical barriers and a lack of functional connectivity in the surroundings, preventing these animals to disperse out. We conclude that South American coati populations living in urban forest fragments of Campo Grande are affected by the epidemiological scenario of VL, known to involve dogs, vectors and humans. We highlight the importance of investigate the parasitism by *L. infantum* in this and other potential *L. infantum* reservoirs that inhabit urbanized regions endemic to VL.

## 1 Introduction

The protozoan *Leishmania infantum* is the etiological agent of visceral leishmaniasis (VL), one of the most important parasitic diseases worldwide. Brazil ranks the top five countries with the highest number of human cases and the highest fatality rate (PAHO, 2020). Despite many control measures for VL in Brazil, the incidence of human cases is still rising, especially in urban areas. Indeed, the country shows an average of 3.404 new cases/100,000 people per year, and 23 out of 27 states notified human VL cases (MS-BR, 2019; MS-BR, 2021), indicating a widespread occurrence of this disease.

The adaptive plasticity of *Lutzomyia longipalpis* complex (the invertebrate vector of *L. infantum*) to urban environments (Salomón et al., 2015), and the presence of other host species than human and domestic dogs (the main vertebrate reservoir) in urban VL endemic areas contribute to the complexity of the enzootic scenarios. There is strong evidence that synanthropic species as rodents, bats and marsupials play a role in the maintenance of *L. infantum* in urban and peri-urban endemic areas in Brazil (Oliveira et al., 2005; Carreira et al., 2012; Lima et al., 2013; de Castro Ferreira et al., 2015; de Rezende et al., 2017; Castro et al., 2020; Vieira et al., 2022).

Wild carnivore species have been reported parasitized by *L. infantum* in Brazil since the half of the 20^th^ century, when the Deane’s couple described the infection in the crab-eating fox (*Cerdocyon thous*) (Deane and Deane, 1955), although the host was inaccurately reported as hoary fox (*Lycalopex vetulus*) (Courtenay et al., 1996). Indeed, crab-eating fox can be considered a putative *L. infantum* reservoir host (Roque and Jansen, 2014), as attested by parasitological assays in different studies (Lainson et al., 1990; Silva et al., 2000; Courtenay et al., 2002; Quinnell and Courtenay, 2009). Nevetheless, the free-living crab-eating fox infected by *L. infantum* seems to be less competent to infect vectors than dogs (Courtenay et al., 2002). Apart from crab-eating fox, the role of other wild Carnivora in the epidemiology of VL is still unknown. It has been recorded the presence of *L. infantum* in intact skin of a captive bush dog (*Speothos venaticus*) (Figueiredo et al., 2008), although the potential of its transmissibility for the vector was reported to be low (Mol et al., 2015). The transmissibility shown for captive maned wolves (*Chrysocyon brachyurus)* was also very low (Mol et al., 2015). As these species do not live near human habitations, they are considered to have a secondary importance in epidemiology of VL in the urban areas (Dantas-Torres and Brandão-Filho, 2006).

The South American coati (Procyonidae: *Nasua nasua*; hereafter “coati”) is a carnivore species well adapted to urban forest fragments in some regions of Brazil (Estevam et al., 2020; Barreto et al., 2021). To date, the only association between *L. infantum* and this *taxon* was reported by Paiz et al. (2015), who showed serological evidence of infection. Additionally, some reports have detected *Leishmania* spp. in this carnivore species through serological and molecular methods (Voltarelli et al., 2009; Porfirio et al., 2018; Reis et al., 2020), and *L. shawi* was isolated from this mammal (Lainson et al., 1989).

Campo Grande (CG), the capital of Mato Grosso do Sul (MS) state, Midwest Brazil, is considered an endemic area for human and canine VL (Botelho and Natal, 2009; Furlan, 2010; Brazuna et al., 2012; de Sousa et al., 2013; SES/MS, 2022). In this region, *L. infantum* has been detected in domestic cats (Antunes et al., 2016; Metzdorf et al., 2017) and wild mammal species as opossum and bats (Humberg et al., 2012; de Rezende et al., 2017; Castro et al., 2020). Moreover, *L. longipalpis* is present in this region (Cunha et al., 2014), indicating a high-risk area for *L. infantum* transmission (Falcão de Oliveira et al., 2020; SES/MS, 2022). Since, Barreto et al. (2021) reported that coati is a conspicuous species in the urban forest fragments at CG, we aimed to investigate the *L. infantum* infection in this mammal population. Our hypothesis is that this wild mammal species is affected by the local epidemiological scenario of *L. infantum* transmission.

## 2 Material and methods

### 2.1 Study areas and field procedures

**Figure 1:**
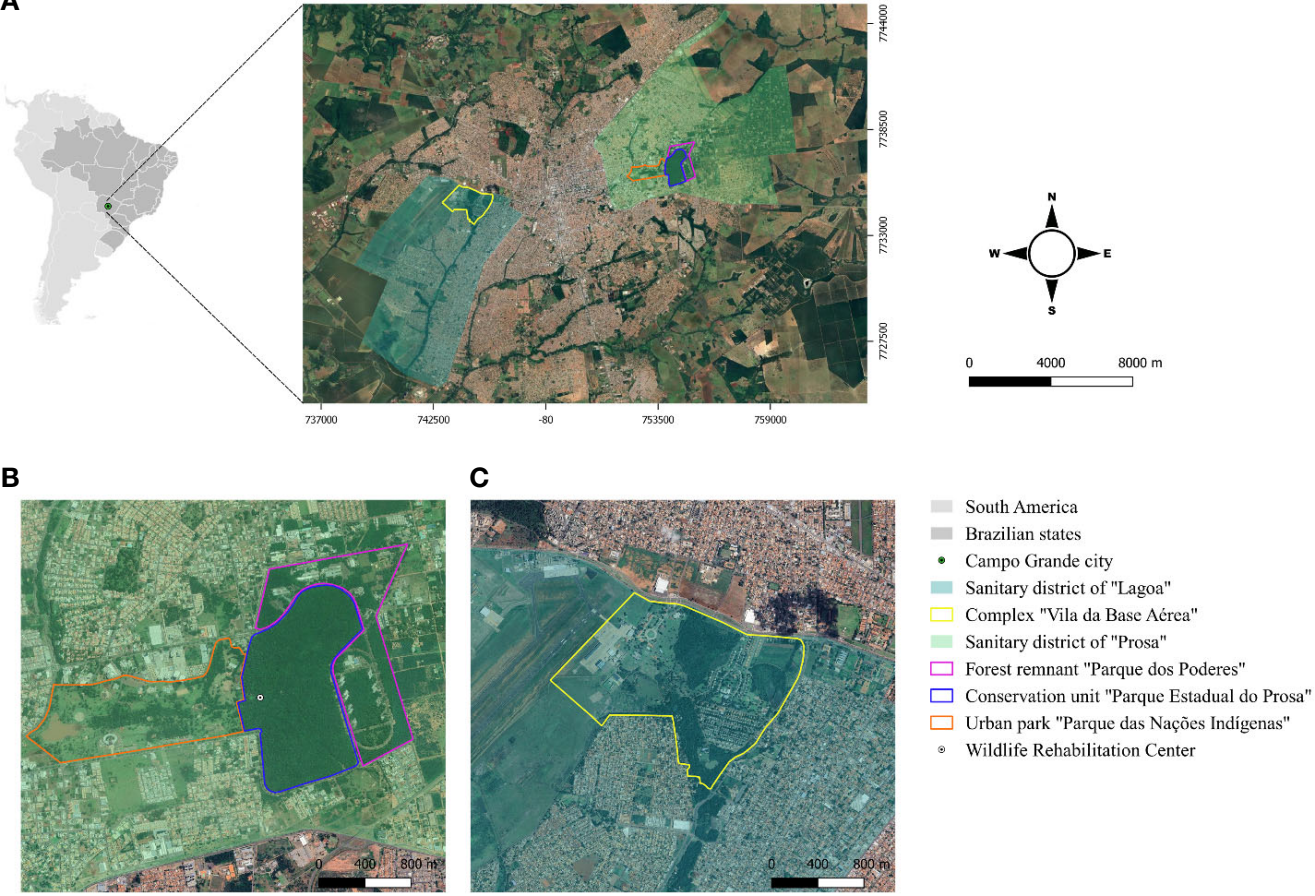
**(A)** Geographical location of the study areas in Campo Grande, Mato Grosso do Sul, Midwest Brazil; **(B)** Conservation unit “Parque Estadual do Prosa” and its adjacent areas “Parque dos Poderes” and “Parque das Naç ões Indígenas”; **(C)** Complex “Vila da Base Aeríea”.

The study was conducted in two forest fragments “*Parque Estadual do Prosa*” (PEP) (20°26’59’’S, 54°33’55’’O) and “*Vila da Base Aérea*” (VBA) (20°28’17’’S, 54°39’14’’O), located in the urban area of CG/MS (**Figure 1A**). The population densities of coatis in these areas were estimated as 11.2 individuals/km^2^ in PEP and 19.5 individuals/km^2^ in VBA (Barreto et al., 2021). The fieldwork occurred every two months from March 2018 to May 2019 encompassing two weeks in each area.

**Figure 2:**
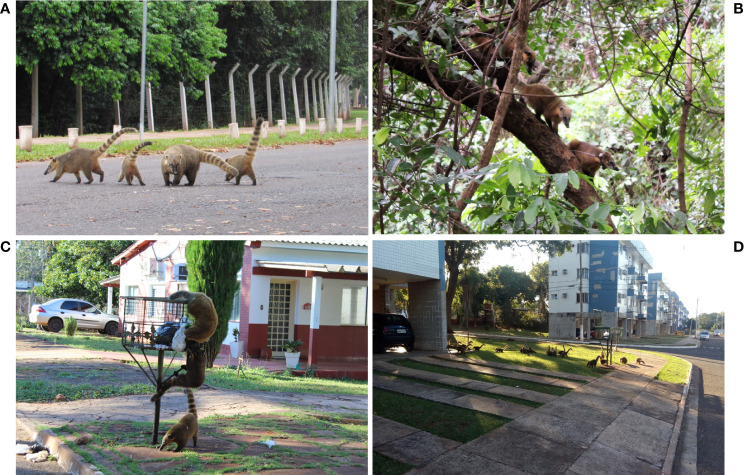
South American coatis (*Nasua nasua*) in the conservation unit “*Parque Estadual do Prosa*” (PEP) and in the residential area “*Vila da Base Aérea*” (VBA) at Campo Grande, Mato Grosso do Sul, Midwest Brazil. **(A)** South American coatis crossing paved streets from “*Parque dos Poderes*” to PEP; **(B)** South American coatis using the arboreal strata to move from PEP to *Parque das Nações Indígenas*; **(C)** South American coatis searching for food in outdoor trash cans in VBA; **(D)** South American coatis foraging near the not fenced houses in VBA.

The PEP is a conservation unit of 135 hectares composed of *cerradão* (tall savanna woodland) and riparian forest, and is located in the sanitary district of “*Prosa*” (**Figure 1B**). Human access is restricted in the PEP, but there is daily flow of people in the adjacent areas, the urban park “*Parque das Nações Indígenas*” and the public administration area “*Parque dos Poderes*” located in a forest remnant of Cerrado. Coatis have free access in all the three areas either by crossing the paved streets or passing through the branches of the trees near the borders (**Figures 2A, B**). In addition, the PEP presents a diverse mammalian fauna and shelters a Wildlife Rehabilitation Center (CRAS) (https://www.imasul.ms.gov.br/centro-de-reabilitacao-de-animais-silvestres-cras/) that receives and keeps several wild animals from all over the MS state.

The VBA, located in the sanitary district of “*Lagoa*”, is a complex of 197 hectares divided into a military operational area and a residential area surrounded by a dense vegetation that includes riparian forest and *veredas* (a plant formation with hydromorphic soil located in the vicinity of river springs) (**Figure 1C**). The residential portion is inhabited by at least 730 humans who raise domestic animals such as dogs, cats and chickens. The houses are not fenced and have many trash cans in front, which serve as food source for the coatis (**Figures 2C, D**). During fieldwork, few individuals of other wild mammal species were seen in the area, as the nine-banded armadillo (*Dasypus novemcinctus*), Azara’s agouti (*Dasyprocta azarae*), Brazilian guinea pig (*Cavia aperea*) and howler-monkey (*Alouatta caraya*), and other species such as the coati and Capybara (*Hydrochoerus hydrochaeris*) are abundant.

The coatis were captured and randomly recaptured in box traps (90 × 45 × 50; Equipos Fauna^®^, Brazil) baited with 15 to 25 grams of bacon. All the captured coatis were sedated with an association of tiletamine hydrochloride and zolazepam hydrochloride (Telazol 100g; Zoetis^®^, USA), tagged with subcutaneous transponders (Animal Tag^®^, Brazil) and numbered colored ear-tags (Qualyplast^®^ Brazil). Blood samples were collected through femoral venipuncture using 0.25 x 0.8 mm needles (BD Vacutainer^®^) and deposited in clot activator tubes to obtain the serum. Bone marrow (BM) samples were collected from manubrium sterni of animals > 2.5 kg using 0.40 x 1.2 mm hypodermic needles and 10 mL syringes, and then placed in tubes containing Ethylenediamine Tetraacetic Acid (EDTA). These procedures were performed after appropriate asepsis of the collection site using bactericide soap, iodized ethanol and ethanol 70%. Ear skin fragments (0.4 to 30 mg) were obtained with sterilized material and stored in 99% ethanol (Sigma-Aldrich^®^, United States). After total recovery from anesthesia, the animals were released at the capture site.

The field procedures were conducted in accordance with the *Instituto Chico Mendes de Conservação da Biodiversidade* (license number 56912-2), the *Instituto de Meio Ambiente de Mato Grosso do Sul* (license number 05/2017, process No.61/405959/2016), and Air force cooperation agreement (No.01/GAP-CG/2018). The present study was approved by the Ethics Committee for Animal Use of Universidade Católica Dom Bosco, Campo Grande, MS (license number 001/2017).

### 2.2 Serological detection of *Leishmania infantum*

**Figure 3:**
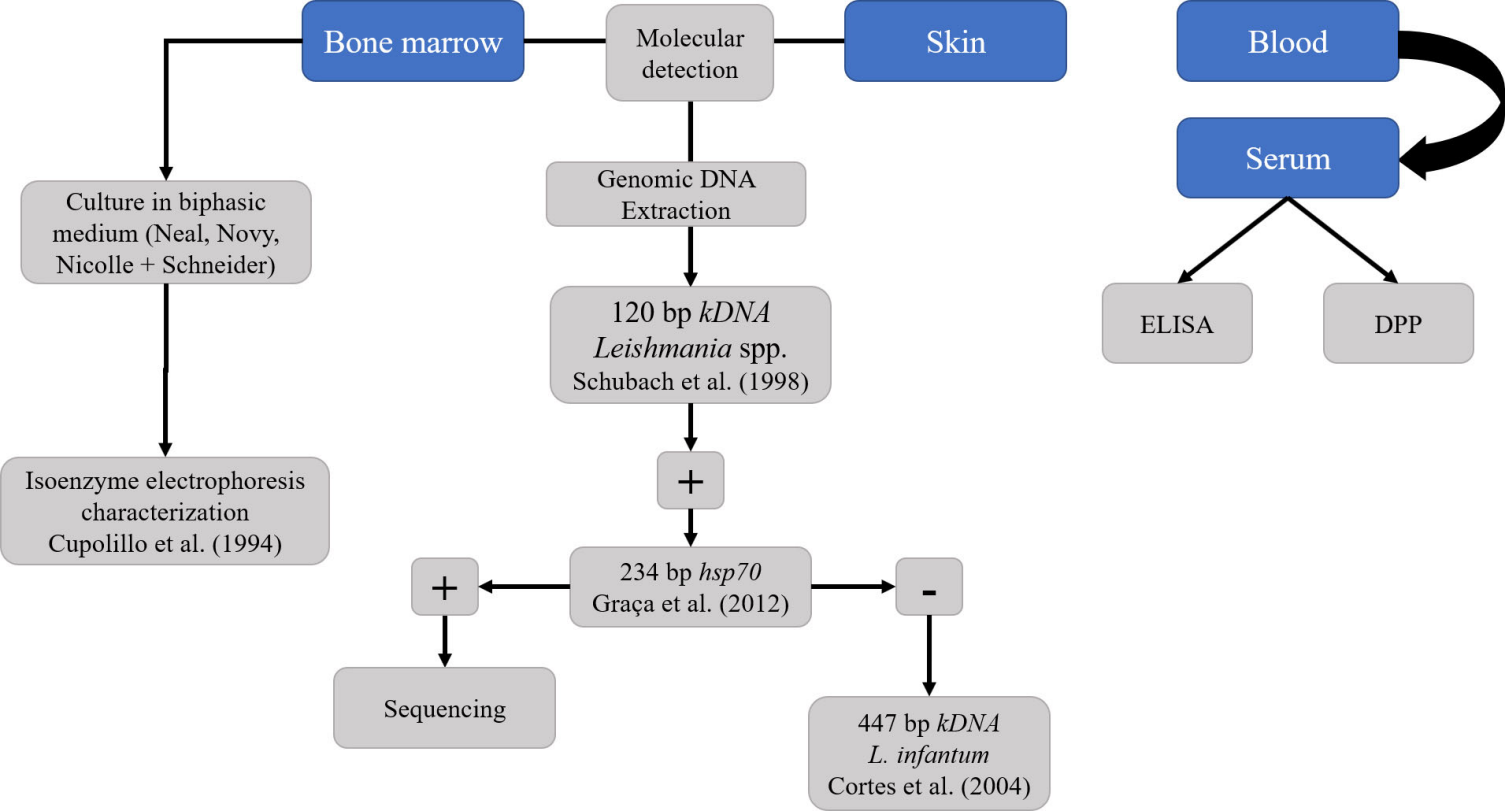
Diagnostic strategy for detection of *Leishmania infantum* in South American coatis (*Nasua nasua*) from forest fragments of Campo Grande, Mato Grosso do Sul, Midwest Brazil.

A serological survey for the detection of anti-*L. infantum* IgG was performed through the Enzyme-Linked Immunosorbent Assay (ELISA) (modified from EIE^®^, BioManguinhos, Rio de Janeiro, Brazil) (Alves et al., 2016) (**Figure 3**). The sera of the coatis were tested with anti-Raccoon IgG HRP conjugated (Bethyl Laboratories^®^, Inc., Montgomery, Texas, United States), diluted at 1:70,000, and each microtiter polystyrene plate contained positive and negative control samples in duplicates. The cut-off point was established by the mean Optical Density (OD) of the negative control ± three standard deviations and the gray range adopted was 20% above the cut-off value. Serum samples were also submitted to a Dual Path Platform chromatographic immunoassay (DPP^®^ CVL BioManguinhos, Rio de Janeiro, Brazil) for rapid detection of K26/K39 specific antibodies against *L. infantum* (da Costa et al., 2003; Rodrigues et al., 2022). To check the reliability of rapid test, the serum samples were tested for Protein A affinity, showing high affinity, as similar as the dog serum samples (data not shown). To improve the accuracy of the serological detection of *L. infantum*, the serum samples of coatis were also screened for *Trypanosoma cruzi* infection using ELISA according to Alves et al. (2016), and the Chagas/Bio-Manguinhos Lateral Flow Immunochromatographic Rapid Test (Chagas-LFRT), as described by Rodrigues et al. (2022). All the tests were performed according to the manufacturer’s recommendations, and seropositivity was considered only when the samples presented positivity in both anti-*L. infantum* IgG ELISA and DPP^®^ CVL.

### 2.3 Parasitological and molecular detection of *Leishmania infantum*

The axenic cultures were performed by placing 200 µL of BM aspirates in duplicates in biphasic media, Novy-MacNeal-Nicolle (NNN) with Schneider’s insect medium overlay supplemented with 10% fetal bovine serum. The tubes were incubated at 26°C – 28°C and weekly examined for 30 days. After the exponential growth phase of the flagellates, the positive culture was cryopreserved at – 196°C under liquid nitrogen. The promastigote forms of the isolated culture were deposited in the Collection of *Leishmania* (CLIOC) (clioc.fiocruz.br) of Instituto Oswaldo Cruz and characterized by them using isoenzyme electrophoresis of G6PDH and 6PGDH (Cupolillo et al., 1994).

Genomic DNA was extracted from 200 µL of the BM samples through the phenol-chloroform method according to Sambrook and Russell (2001), and from the skin fragments using the DNeasy^®^ Blood and Tissue Kit (Qiagen^®^, Netherlands). The DNA was eluted in 50 µL elution buffer and stored at -20°C until molecular analysis. The concentration and quality were assessed through spectrophotometry (Biodrop, Analítica^®^, Brazil).

The BM and skin samples were firstly screened through Polymerase Chain Reaction (PCR) using a pair of primers (L1/L2) directed to the kinetoplastid DNA (kDNA) minicircle molecules of *Leishmania* spp. according to Schubach et al. (1998). Positive samples were then submitted to PCR targeting the heat-shock protein 70 gene (HSP70) and sequenced, as described by Graça et al. (2012) (**Figure 3**). Sanger sequencing was performed in the AB3500 platform (Applied Byosystems^®^, United States) at ACTGene Molecular Analysis biotechnology company (https://actgene.com.br/). Negative samples at HSP70 PCR were submitted to *L. infantum*-specific kDNA PCR (MC1/MC2) according to Cortes et al. (2004) (**Figure 3**). All the PCRs were conducted in a thermocycler (GeneAmp PCR System 9600, Applied Byosystems^®^, United States), using positive controls of *L. infantum* (LHV14 strain) and *L. amazonensis* (IFLA/BR/1967/PH8 strain) and negative controls (ultrapure water). The PCR products were visualized on 1.5% agarose (Kasvi^®^, Brazil), stained with Gel Red Nucleic Acid Stain (Biotium^®^, EUA).

### 2.4 Statistical analysis

In order to observe the agreement between serological assays (ELISA and DPP^®^ CVL), the Cohen’s Kappa test was performed with a 95% confidence interval and interpreted as follows: poor agreement (k = 0), slight agreement (0.20 ≤ k ≥ 0), fair agreement (0.40 ≤ k ≥ 0.21), moderate agreement (0.6 ≤ k ≥ 0:41), substantial agreement (0.80 ≤ k ≥ 0.61), and almost perfect agreement (1.0 ≤ k ≥ 0.81). To check a possible influence of the studied areas and sex on *L. infantum* infection, a Chi-squared test (p < 0.05) was applied considering the individuals. We considered infection by *L. infantum* as positivity in any of the parasitological or molecular tests and/or positivity in both serological assays. Both Cohen’s Kappa and Chi-squared tests were performed through the R software (R Development Core Team, 2015).

## 3 Results

### 3.1 Captures, recaptures and acquisition of samples

During the study, a total of 110 coatis were captured in the VBA and PEP. Blood was collected in all capture occasions corresponding to 21 males and 30 females from PEP, and 25 males and 34 females from VBA. The BM samples were collected from 85 individuals corresponding to 16 males and 26 females from PEP, and 19 males and 24 females from VBA. Skin samples were collected from 65 ear-tagged animals corresponding to 10 males and 20 females from PEP, and 14 males and 21 females from VBA.

**Figure 4:**
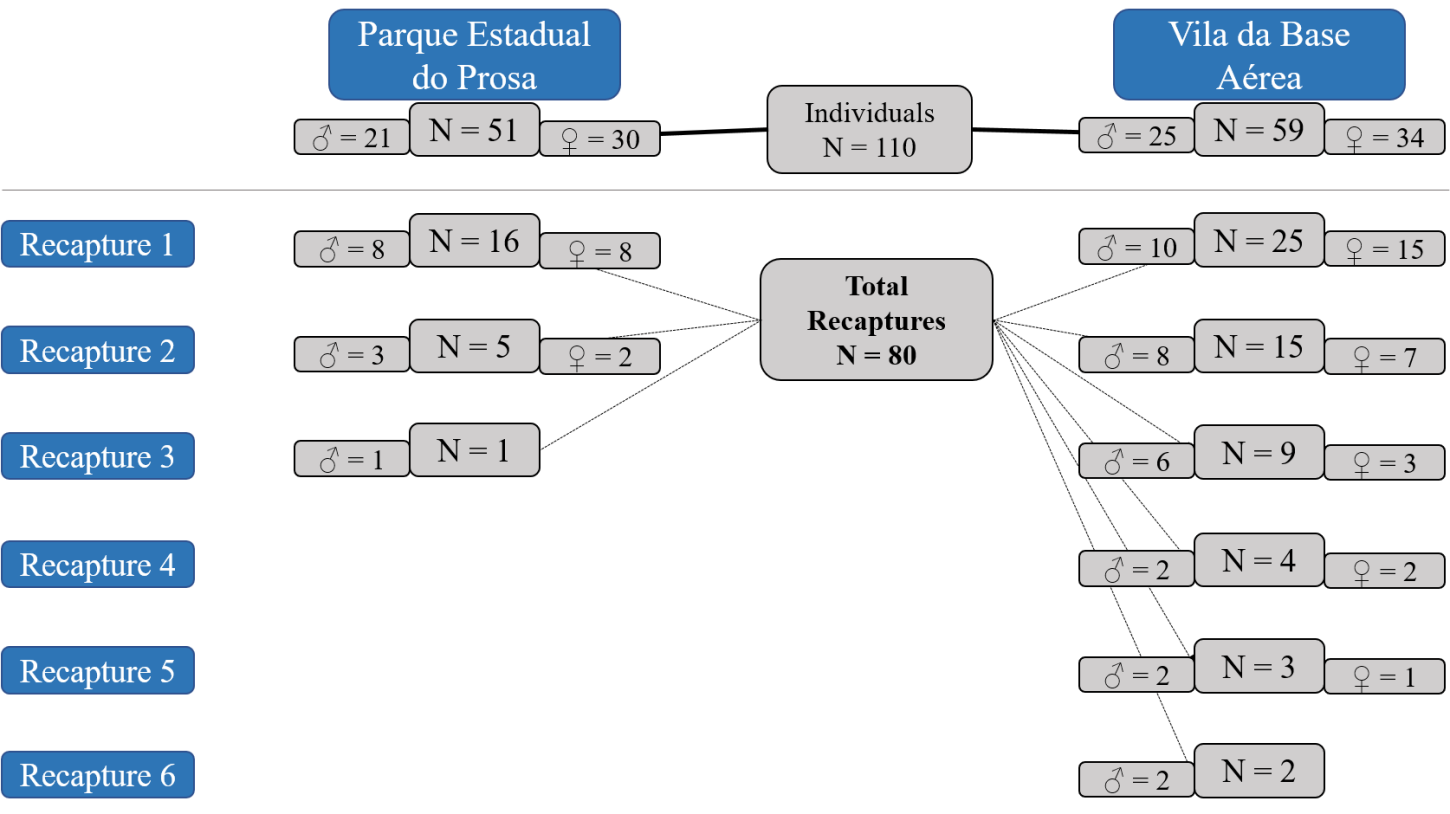
Recapture events of South American coatis (*Nasua nasua*) from the conservation unit “*Parque Estadual do Prosa*” (PEP) and the residential area “*Vila da Base Aérea*” (VBA) from March 2018 to April 2019.

Throughout the study, we recaptured a total of 41 individuals (eight males and eight females from PEP, and 10 males and 15 females from VBA) in 80 occasions (**Figure 4**). Serum samples were collected, and serologic tests were performed in all recaptures, totalizing 190 samples (73 from PEP and 117 from VBA). Bone marrow samples were collected from 51 recaptures and submitted to molecular analysis (totalizing 136 samples = 63 from PEP and 73 from VBA). Skin samples were not collected during recaptures.

### 3.2 Serological detection of *Leishmania infantum*

A total of 65 samples from 33 individuals were seropositive (ELISA+/DPP+) to *L. infantum*, indicating an overall seroprevalence of 30% (33/110). In PEP, the seroprevalence rate was 11.8% (6/51), corresponding to two males and four females, while in VBA the seroprevalence was estimated in 45.8% (27/59), corresponding to 11 males and 16 females. Despite the discordance observed in 19 samples (16 ELISA+/DPP- and three ELISA-/DPP+), the Cohen’s kappa test attested a substantial agreement between ELISA and DPP (*k* = 0.78; *CI* 95% = 0.68 – 0.87; *p* = 0.001). Only one serum sample of a non-recaptured female coati from VBA, that was seropositive to *L. infantum*, presented seropositivity for *T. cruzi*, indicating a mixed infection.

### 3.3 Parasitological and molecular detection of *Leishmania infantum*

**Figure 5:**
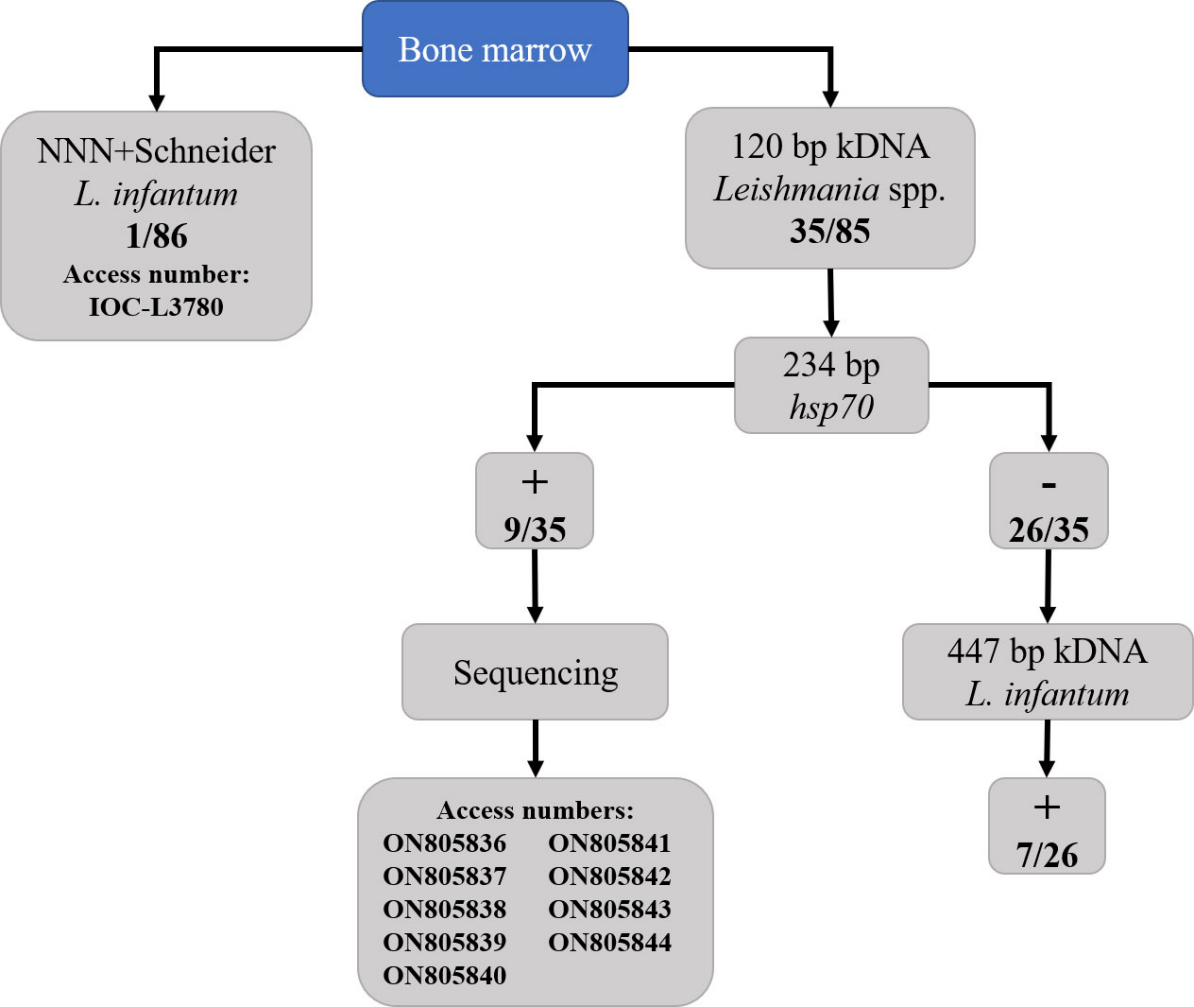
Parasitological and molecular detection of *Leishmania infantum* from the bone marrow samples of South American coatis (*Nasua nasua*) from urban forest fragments in Campo Grande, Mato Grosso do Sul, Midwest Brazil.

During our study, we isolated *L. infantum* from one BM sample derived from a seropositive male captured in VBA (ID 11528) (**Figure 5**). This isolate was deposited in the CLIOC (access number: IOC-L 3780), but the molecular diagnosis was not performed in this individual due to insufficient amount of BM sample.

The screening kDNA PCR in BM samples showed an infection rate of 41.2% (35/85) for *Leishmania* sp. Posteriorly, *L. infantum* infection was confirmed in 16 individuals (nine by HSP70 sequencing and seven by MC1/MC2 PCR), seven from PEP (four males and three females) and nine from VBA (three males and six females). The results of parasitological and molecular assays performed in the coatis are presented in **Figure 5**. From the 19 individuals negative to *L. infantum* in molecular tests, four were classified as seropositive and 15 were seronegative. Only one skin sample was positive in kDNA PCR and *L. infantum* infection was confirmed by MC1/MC2 PCR. The same individual (ID 10826, a male from VBA) was seropositive and also positive in BM molecular assay (with *L. infantum* infection confirmed by HSP70 sequencing).

The BLASTn analysis of the nine HSP70 DNA sequences revealed identity of 100% with *L. donovani* HSP70 gene obtained from samples of humans from Ethiopia and China (accession numbers: FN669773.1 and JX970996.1 respectively). The obtained sequences were deposited in the GenBank database under the following accession numbers: ON805836 to ON805844 (**Figure 5**).

### 3.4 Data analysis

Together, the parasitological, serology and molecular tests showed a *L. infantum* infection rate of 36.4% (40/110). These 40 individuals correspond to 24 positive by serological tests (including an individual positive in parasitological test), seven positive by molecular tests, and nine positive by both tests. We observed through Chi-squared test that infection was significantly higher in VBA (49.1%, 29/59) than in PEP (21.6%, 11/51) (*Chi^2^* = 90; *df* = 1; *p* = 0.003). Our analysis did not show influence of sex in *L. infantum* infection (*Chi^2^* = 1.580; *df* = 1; *p* = 0.208).

When considering only individuals assessed by both serology and molecular tests (N = 85), we observed distinct *L. infantum* infection patterns (**Table 1**).

**Table 1:** South American coatis (*Nasua nasua*) infected by *Leishmania infantum* by both serological and molecular diagnosis at urban forest fragments in Campo Grande, Mato Grosso do Sul, Brazil.

Areas	Serology + Molecular -	Serology -Molecular +	Serology + Molecular +	Total
PEP	4/42 (9.5%)	5/42 (11.9%)	2/42 (4.8%)	11/42 (26.2%)
VBA	16/43 (37.2%)	2/43 (4.6%)	7/43 (16.3%)	25/43 (58.1%)
Total	20/85 (23.5%)	7/85 (8.2%)	9/85 (10.6%)	36/85 (42.3%)

PEP: conservation unit “Parque Estadual do Prosa”; VBA: residential área “Vila da Base Aérea”. Four individuals from VBA (two male and two female) were excluded from this table because no molecular assay was performed.

### 3.5 Longitudinal survey

**Figure 6:**
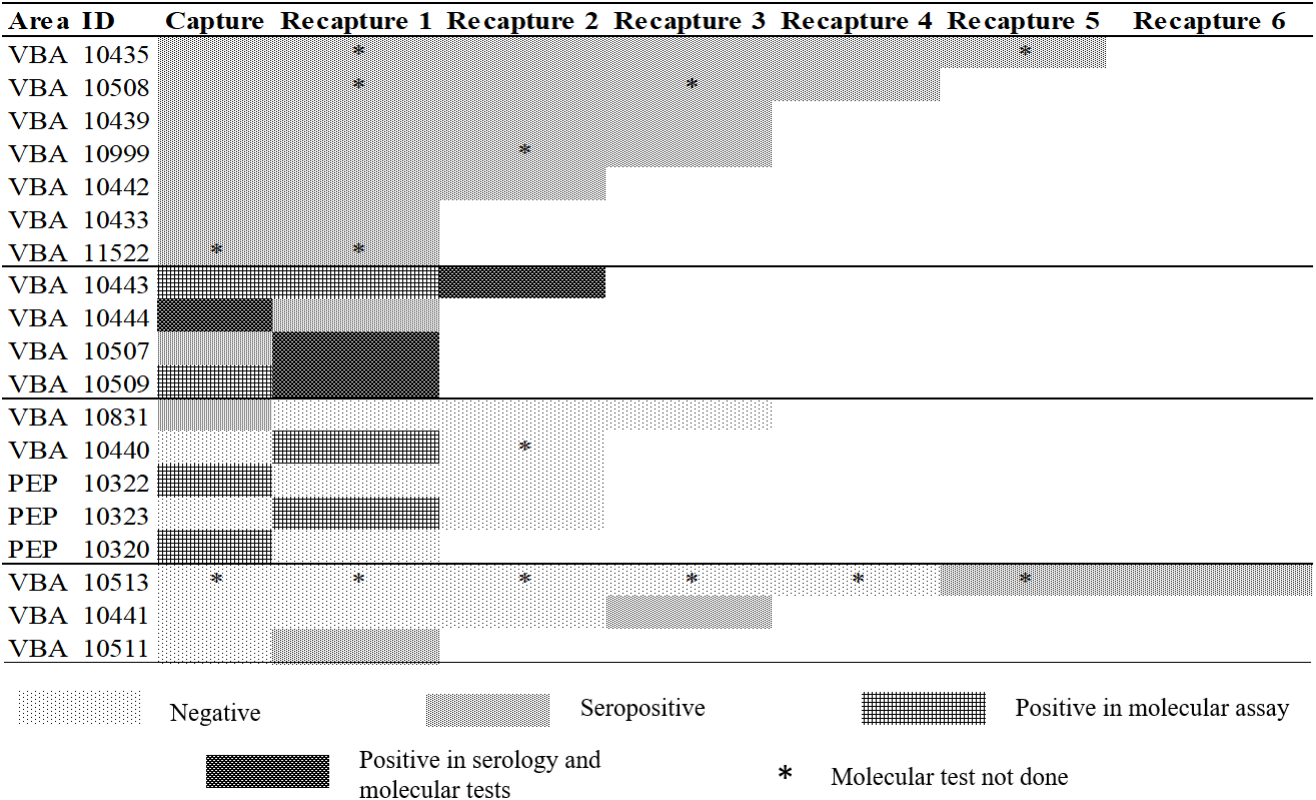
Longitudinal results of 19 South American coatis (*Nasua nasua*) infected by *Leishmania infantum* in urban forest fragments in Campo Grande, Mato Grosso do Sul, Midwest Brazil, from March 2018 to April 2019. ID: Identification number; PEP: conservation unit *Parque Estadual do Prosa*; VBA: residential rea *Vila da Base Aérea*. Positivity in molecular was obtained either by HSP70 sequencing or MC1/MC2 PCR. Seropositivity was considered when both ELISA and DPP^®^ CVL tests were positive.

Among the 40 coatis positive for *L. infantum*, 19 individuals were recaptured during the study at least on one occasion. Eleven individuals remained positive by at least one diagnostic test throughout their recaptures (**Figure 6**). Of these, seven were only seropositive (IDs 10433, 10435, 10439, 10442, 10508, 10999 and 11522) and four presented positivity also in molecular tests (IDs 10443, 10444, 10507 and 10509). Five individuals (IDs 10320, 10322, 10323, 10440 and 10831) presented positivity (four by MC1/MC2 PCR and one by serological test) either in the capture or in the first recapture, and negativity in the subsequent recapture (**Figure 6**). We observed three seroconversion events, with individuals (IDs, 10441, 10511 and 10513) presenting positivity only in the subsequent recaptures (**Figure 6**). The remaining twenty-one positive animals were not recaptured: 13 were seropositive, five were positive in both serology and molecular tests and three were positive only in molecular assays.

## 4 Discussion

In this study we report serological, parasitological and molecular detection of *L. infantum* in coatis from forested fragments of an endemic region for VL, with an overall infection rate of 36.4% (40/110). Our results demonstrate that this wild carnivore species is somehow affected by the *L. infantum* epidemiological scenario at the studied area. Indeed, (i) the endemicity of VL in humans and dogs at CG (SES/MS, 2022), (ii) abundance of the main vector *L. longipalpis* s.l. (de Souza Fernandes et al., 2022), and (iii) the high density of coatis in forest fragments of CG (Barreto et al., 2021) may favor the *L. infantum* transmission to coatis. Future studies may indicate the genetic flow of *L. infantum* populations among the multiple hosts involved in this epidemiological scenario, which was not the aim of the present study. The evaluation of recaptured individuals was a particular aspect of the present study because it provided evidence of long-term exposure of *L. infantum* in the studied populations, including seroconversion events.

Our findings indicated that *L. infantum* infect coatis from both studied forest fragments in CG, but a higher rate of infection in VBA when compared to PEP was observed (p = 0.003), indicating a spatial influence. This finding may be related to the geographical localization of the studied areas, as well as differences in land use. For instance, VBA is a residential area with many dogs, the main domestic reservoir host of *L. infantum*, and chickens, which although refractory to *Leishmania* infection, are amplifiers and attractors of sandfly populations (Casanova et al., 2013). Even though maximal dispersion of *L. longipalpis* is reported to be 243 meters in urban areas (de Oliveira et al., 2013), Coatis at VBA often forage near houses looking for food in outdoor trash cans, which may favor their exposition to with *L. longipalpis* in this environment. As described by Barreto et al. (2021), VBA has a denser population of coatis than PEP, and in this area the animals are prevented from dispersing out due to the physical barrier and the lack of functional connectivity in the surroundings. In addition, VBA is in the sanitary district of “*Lagoa*”, a region that had the higher number of notified cases and cumulative incidence of human VL from 2002 to 2009 (Brazuna et al., 2012). This sanitary district also had the second highest number of canine VL from 2007 to 2009 in CG, and a large sandfly abundance (Brazuna et al., 2012; de Souza Fernandes et al., 2022).

Oppositely, the lower infection rate observed in PEP (21.6%) may be related to several factors including the absence or low abundance of competent reservoirs, a higher richness of mammal species than VBA, and the CRAS, that is located in this conserved area. Although the coatis have access to the adjacent areas *Parque das Nações Indígenas* and *Parque dos Poderes*, there are no resident dogs in these areas that could sustain high rates of *L. infantum* infection, as in VBA. Also, there are 17 mammal species listed in the PEP Management Plan, and this number can be higher if we consider the captive animals of the CRAS, while in VBA few species were sighted. Assuming that the PEP presents a higher diversity of wild mammal species in comparison to VBA, we can expect that the PEP has a higher number of hosts not competent to be source of infection for the vector, which could reduce the risk of *L. infantum* infection in coatis through dilution effect (Keesing and Ostfeld, 2021). In addition, the CRAS, located inside the PEP, may influence the distribution of *L. longipalpis* due to the constant presence of captive animals and large amount of organic material from feces and concentrate food that could attract the vector and avoid its dispersion.

The detection of *L. infantum* in an ear skin fragment of an individual that was also seropositive and presented *L. infantum* DNA in BM sample (ID 10826), indicates that *L. infantum* is capable to colonize intact skin of coatis. In view of the importance for *L. infantum* transmission, domestic dogs are the main reservoir species mainly because they can present high parasitic loads in the skin, favoring the infection of the vectors (Dantas-Torres, 2007). Wild carnivores, as maned wolf, bush dog and crab-eating fox have been reported presenting *L. infantum* DNA in skin (Figueiredo et al., 2008; Souza et al., 2010; Jusi et al., 2011), although the first two demonstrated low competence to transmit the parasite to the vectors. Since the maintenance of a multi-host parasite as *L. infantum* in a given environment can involve different mammal species with diverse epidemiological competences (Roque and Jansen, 2014), efforts toward evaluation of blood meal source of sand flies in the forest fragments of CG, as well as isolation and quantification of parasite load from skin of different mammal species must be considered. Our results put the coati together with maned wolf and bush dog as carnivore species that may harbor parasite in intact skin. Nevertheless, their role in *L. infantum* epidemiology remains to be uncovered.

Our data showed no significant differences related to sex in the infection rate of *L. infantum* infection. The sex-related response to *L. infantum* infection includes a complex interaction among host, vector, and environment (Lockard et al., 2019), and therefore the sex differences in the prevalence of VL may not have a pattern. While higher VL prevalence in males have been recorded in humans and dogs naturally and experimentally infected (Guerra-Silveira and Abad-Franch, 2013; Lockard et al., 2019), studies with long-term data have shown no differences in the seroprevalence of *L. infantum* infection between male and female stray dogs (Miró et al., 2007; Müller et al., 2022). Risueño et al. (2018) found only a marginally greater *L. infantum* prevalence in female compared to male free-living rabbits. Given the nature of our data, we could not discriminate whether the absence of sex difference in the infection rate of *L. infantum* was due to biological differences or sex-related exposure. However, we can presume that social behavior of coatis in the urban matrix can contribute to a similar rate of infection between male and female. Coatis form social groups consisting of adult females and their offspring, with adult males joining the group only in the reproductive period (Gompper and Decker, 1998). However, Barreto et al. (2021) observed that males are cohesive to the group regardless of the reproductive period.

Our study showed that many individuals presented distinct infection patterns concerning their results in the serological and molecular assays. This dynamic, as well events of new infections observed in the longitudinal studies are expected in populations in endemic areas because individuals are constantly being exposed to infected vectors (Herrera et al., 2004; Oliva et al., 2006). As observed, seven seronegative coatis displayed positivity in the molecular assays suggesting recent infections, a common phenomenon found in populations inhabiting endemic areas (Herrera et al., 2004; Oliva et al., 2006). In dogs, the humoral response may take around three to five months after *L. infantum* infection to produce sufficient immunoglobulins to be detected by serological tests (Paranhos-Silva et al., 2003). It is important to mention that in endemic areas for leishmaniasis, about one fifth of *L. infantum* infected dogs presents low or undetectable anti-*Leishmania* antibodies (Ashford et al., 1995). On the contrary, seropositive animals with negative molecular results are expected because the serological assays detect antibodies that are maintained in serum for longer periods, while the molecular assays are positive only when parasites are present in the specific amount of analyzed sample. We highlight the importance of combine different diagnostic tests in order to improve the detectability of infected individuals.

The molecular diagnostic strategy used here allowed us to detect a greater number of positive samples. In fact, as the sensitivity of the HSP70 gene target is low when compared to the kDNA PCR assay (Graça et al., 2012), and the use of MC1/MC2 (*L. infantum* kDNA), as it is more sensitive and highly specific for *L. donovani* s.l., improved the detectability of molecular diagnostics. Concerning the recaptures, molecular negativity observed in the first recaptures of the individuals 10320 and 10322, and in the second recapture of the individual 10323 (**Figure 6**) could be attributed to fluctuations in detectable DNA from the bone marrow. Transient PCR bone marrow positivity has been reported in dogs naturally and experimentally infected with *L. infantum* (Paranhos-Silva et al., 2003; Oliva et al., 2006). As discussed by these authors, this may also be attributed to limited sensitivity of specific molecular tests, in our case, MC1/MC2 and HSP70, especially in the first and second recaptures of the individuals 10320 and 10323 respectively, which were positive in the screening kDNA for *Leishmania* spp. A rare case of seroreversion was observed in one individual (10831) that was seropositive in the capture and seronegative in the three subsequent recaptures (**Figure 6**). Seroreversion was already reported in both canine and human VL (Ostyn et al., 2011; Maia et al., 2020), probably by decrease of antibody concentration over time, as observed in the ELISA OD from this individual (Capture: 0,168; First recapture: 0,149; Second recapture: 0,104; Third recapture: 0,075).

In contrast, we found evidence of active transmission of *L. infantum* in the area, evidenced by new infections occurring in coatis during the study period. Indeed, five individuals were seronegative in the capture or in a given recapture, and positive by serology or molecular diagnosis in subsequent recapture. Two individuals (IDs 10443 and 10509) remained positive in molecular test in consecutive captures, indicating that individuals may be capable of sustain BM parasitism for at least five months. At this point, the remaining question is whether these animals are capable of sustain high parasite loads in the skin and act as source of infection to sand flies.

Our results point to the occurrence of other *Leishmania* species besides *L. infantum* in the sampled coatis, since (i) 54.3% (19/35) of the infections detected by kDNA PCR were not confirmed by *L. infantum-*specific targets, and (ii) we found 16 samples ELISA positive and DPP negative that were also negative in the serological tests performed to detect other common trypanosomatids that infects wild mammals: *T. cruzi*. Geographical overlap among *Leishmania* species is reported (Talmi-Frank et al., 2010), and probably occurs in Brazil, which shelters the largest richness of *Leishmania* species among Latin American countries (Herrera et al., 2020). In Campo Grande municipality, Nantes et al. (2021) obtained DNA sequences with high similarity to *L. amazonensis* and *L. guyanensis* from blood samples of opossums, and Castro et al. (2020) reported *L. braziliensis* infection in the bats *Lasiurus cinereus* and *Cynomops planirostris*. In addition, the presence of other species of *Leishmania* than *L. infantum* circulating in coatis of Campo Grande is possible due to the occurrence of many phlebotomine species as *Nyssomyia whitmani*, *Ny. antunesi* and *Bichromomyia flaviscutellata*, that are involved in transmission of several species of *Leishmania* commonly reported in outbreaks of cutaneous leishmaniasis such as *L*. *braziliensis*, *L*. *shawi*, *L*. *guyanensis*, *L. lindenbergi* and *L*. *amazonensis* (Costa et al., 2007; Silva et al., 2008; Oliveira et al., 2012; Vásquez Trujillo et al., 2013; Carvalho et al., 2018; de Souza Fernandes et al., 2022).

## 5 Conclusion

Coatis living in urban forested fragments at CG are affected by the epidemiological scenario of VL transmission that evolves dogs, humans and *L. longipalpis* vectors, as demonstrated by their overall infection rate of 36.4%. The importance of this finding is highlighted by: (i) the high population densities of this mammal host in different urban forest fragments, (ii) their proximity to domiciliated areas and contact with human and dogs, (iii) the ability to maintain viable parasites in bone marrow, as demonstrated by *L. infantum* isolation and (iv) the possibility to maintain parasites in no lesioned ear skin, as demonstrated by *L. infantum* DNA detection. Given these findings, we highlight the importance to investigate the pattern of *L. infantum* infection in coatis inhabiting urban forest fragments to define their role as *L. infantum* reservoirs in endemic areas for VL.

## Data availability statement

The datasets presented in this study can be found in online repositories. The names of the repository/repositories and accession number(s) can be found in the article.

## Ethics statement

The animal study was reviewed and approved by Ethics Committee for Animal Use of Universidade Católica Dom Bosco, Campo Grande, MS (license number 001/2017).

## Author contributions

GCdeM, WTGB, ALRR and HMH wrote the manuscript. GCdeM, WTGB, ACR and WOdeA collected biological samples. SCdasCX, FMA and ARdaS contributed in diagnostic tests execution. FMS performed statistical analysis. CEdeO, GEdeOP, SCdasCX, FMA, GBdeA, AMJ, ALRR and HMH critically reviewed the manuscript. All authors contributed to the article and approved the submitted version.

## References

[B1] Alves F. M. de Lima J. S. Rocha F. L. Herrera H. M. Mourão G. de M.. (2016). Complexity and multi-factoriality of *Trypanosoma cruzi* sylvatic cycle in coatis, *Nasua nasua* (Procyonidae), and triatomine bugs in the Brazilian pantanal. Parasit Vectors. 9 (1), 378. doi: 10.1186/s13071-016-1649-4 27370106 PMC4930594

[B2] Antunes T. R. Peixoto R. A. V. Oliveira B. B. Sorgatto S. do Nascimento Ramos C. A. de Souza A. I. (2016). Detection of *Leishmania infantum* in peripheral blood smear and lymph node of a domestic feline. Acta Sci. Vet. 44 (Suppl 1), 162. doi: 10.22456/1679-9216.83208

[B3] Ashford D. A. Bozza M. Freire M. Miranda J. C. Sherlock I. Eulalio C.. (1995). Comparison of the polymerase chain reaction and serology for the detection of canine visceral leishmaniasis. Am. J. Trop. Med. Hyg. 53, 251–255. doi: 10.4269/ajtmh.1995.53.251 7573707

[B4] Barreto W. T. G. Herrera H. M. de Macedo G. C. Rucco A. C. de Assis W. O. Oliveira-Santos L. G.. (2021). Density and survivorship of the south American coati (*Nasua nasua*) in urban areas in central–Western Brazil. Hystrix It. J. Mamm. 32 (1), 82–88. doi: 10.4404/hystrix-00386-2020

[B5] Botelho A. C. Natal D. (2009). First epidemiological description of visceral leishmaniasis in campo grande, state of mato grosso do sul. Rev. Soc Bras. Med. Trop. 42 (5), 503–508. doi: 10.1590/s0037-86822009000500006 19967231

[B6] Brazuna J. C. Silva E. A. Brazuna J. M. Domingos I. H. Chaves N. Honer M. R.. (2012). Profile and geographic distribution of reported cases of visceral leishmaniasis in campo grande, state of mato grosso do sul, Brazil, from 2002 to 2009. Rev. Soc Bras. Med. Trop. 45 (5), 601–606. doi: 10.1590/s0037-86822012000500012 23152344

[B7] Carreira J. C. da Silva A. V. de Pita Pereira D. Brazil R. P. (2012). Natural infection of *Didelphis aurita* (Mammalia: Marsupialia) with *Leishmania infantum* in Brazil. Parasitol. Vectors. 5, 111. doi: 10.1186/1756-3305-5-111 PMC3409019 22676324

[B8] Carvalho B. M. dos Santos T. V. da R. Barata I. Lima J. A. N. Silveira F. T. Vale M. M.. (2018). ). entomological surveys of *Lutzomyia flaviscutellata* and other vectors of cutaneous leishmaniasis in municipalities with records of *Leishmania amazonensis* within the bragança region of pará state, brazil. J. Vector Ecol. 43 (1), 168–178. doi: 10.1111/jvec.12296 29757525

[B9] Casanova C. Andrighetti M. T. Sampaio S. M. Marcoris M. L. Colla-Jacques F. E. Prado A. P. (2013). Larval breeding sites of *Lutzomyia longipalpis* (Diptera: Psychodidae) in visceral leishmaniasis endemic urban areas in southeastern Brazil. PloS Negl. Trop. Dis. 7 (9), e2443. doi: 10.1371/journal.pntd.0002443 24069494 PMC3777886

[B10] Castro L. S. Dorval M. E. C. Matheus L. M. D. Bednaski A. V. Facco G. G. Silveira M.. (2020). *Leishmania* presence in bats in areas endemic for leishmaniasis in central-west Brazil. Int. J. Parasitol: Parasites Wildl. 11, 261–267. doi: 10.1016/j.ijppaw.2020.02.008 32195111 PMC7078454

[B11] Cortes S. Rolão N. Ramada J. Campino L. (2004). PCR as a rapid and sensitive tool in the diagnosis of human and canine leishmaniasis using leishmania donovani s.l.-specific kinetoplastid primers. Trans. R. Soc Trop. Med. Hyg. 98 (1), 12–17. doi: 10.1016/s0035-9203(03)00002-6 14702834

[B12] Costa S. M. Cechinel M. Bandeira V. Zannuncio J. C. Lainson R. Rangel E. F. (2007). *Lutzomyia* (*Nyssomyia*) whitmani s.l. (Antunes & coutinho 1939) (Diptera: Psychodidae: Phlebotominae): geographical distribution and the epidemiology of American cutaneous leishmaniasis in Brazil – mini review. Mem. Inst. Oswaldo Cruz. 102, 149–153. doi: 10.1590/s0074-02762007005000016 17426877

[B13] Courtenay O. Quinnell R. J. Garcez L. M. Dye C. (2002). Low infectiousness of a wildlife host of *Leishmania infantum*: the crab-eating fox is not important for transmission. Parasitol 125 (Pt 5), 407–414. doi: 10.1017/s0031182002002238 12458824

[B14] Courtenay O. Santana E. W. Johnson P. J. Vasconcelos I. A. Vasconcelos A. W. (1996). Visceral leishmaniasis in the hoary zorro *Dusicyon vetulus*: a case of mistaken identity. Trans. R. Soc Trop. Med. Hyg. 90 (5), 498–502. doi: 10.1016/s0035-9203(96)90293-x 8944254

[B15] Cunha R. C. Andreotti R. Cominetti M. C. Silva E. A. (2014). Detection of *Leishmania infantum* in *Lutzomyia longipalpis* captured in campo grande, MS. Rev. Bras. Parasitol. Vet. 23 (2), 269–273. doi: 10.1590/S1984-29612014049 25054512

[B16] Cupolillo E. Grimaldi G. Jr Momen H. (1994). A general classification of new world *Leishmania* using numerical zymotaxonomy. Am. J. Trop. Med. Hyg. 50 (3), 296–311. doi: 10.4269/ajtmh.1994.50.296 8147488

[B17] da Costa R. T. França J. C. Mayrink W. Nascimento E. Genaro O. Campos-Neto A. (2003). Standardization of a rapid immunochromatographic test with the recombinant antigens K39 and K26 for the diagnosis of canine visceral leishmaniasis. Trans. R. Soc Trop. Med. Hyg. 97 (6), 678–682. doi: 10.1016/s0035-9203(03)80102-5 16117962

[B18] Dantas-Torres F. (2007). The role of dogs as reservoirs of *Leishmania* parasites, with emphasis on *Leishmania* (*Leishmania*) *infantum* and *Leishmania* (*Viannia*) *braziliensis* . Vet. Parasitol. 149, 139–146. doi: 10.1016/j.vetpar.2007.07.007 17703890

[B19] Dantas-Torres F. Brandão-Filho S. P. (2006). Visceral leishmaniasis in Brazil: revisiting paradigms of epidemiology and control. Rev. Inst. Med. Trop. Sao Paulo. 48 (3), 151–156. doi: 10.1590/s0036-46652006000300007 16847505

[B20] Deane L. M. Deane M. P. (1955). Observações preliminares sobre a importância comparativa do homem, do cão e da raposa (*Lycalopex vetulus*) como reservatórios da *Leishmania donovani* em áreas endêmicas de calazar, no ceará. Hospital 48, 79–98.

[B21] de Castro Ferreira E. Cruz I. Cañavate C. de Melo L. A. Pereira A. A. Madeira F. A.. (2015). Mixed infection of *Leishmania infantum* and *Leishmania braziliensis* in rodents from endemic urban area of the new world. BMC Vet. Res. 11, 71. doi: 10.1186/s12917-015-0392-y 25890323 PMC4374209

[B22] de Oliveira E. F. Silva E. A. Casaril A. E. Fernandes C. E. Paranhos Filho A. C. Gamarra R. M.. (2013). Behavioral aspects of *Lutzomyia longipalpis* (Diptera: Psychodidae) in urban area endemic for visceral leishmaniasis. J. Med. Entomol. 50 (2), 277–284. doi: 10.1603/me12082 23540114

[B23] de Rezende M. B. Herrera H. M. Carvalho C. M. E. Carvalho Anjos E. A. Ramos C. A. N. de Araújo F. R.. (2017). Detection of leishmania spp. in bats from an area of Brazil endemic for visceral leishmaniasis. Transbound Emerg. Dis. 64 (6), e36–e42. doi: 10.1111/tbed.12597 28233434

[B24] de Sousa K. C. André M. R. Herrera H. M. de Andrade G. B. Jusi M. M. dos Santos L. L.. (2013). Molecular and serological detection of tick-borne pathogens in dogs from an area endemic for *Leishmania infantum* in mato grosso do sul, Brazil. Rev. Bras. Parasitol. Vet. 22 (4), 525–531. doi: 10.1590/S1984-29612013000400012 24473877

[B25] de Souza Fernandes W. de Oliveira Moura Infran J. Falcão de Oliveira E. Etelvina Casaril A. Petilim Gomes Barrios S. Lopes de Oliveira S. L.. (2022). Phlebotomine sandfly (Diptera: Psychodidae) fauna and the association between climatic variables and the abundance of *Lutzomyia longipalpis* sensu lato in an intense transmission area for visceral leishmaniasis in central Western Brazil. J. Med. Entomol. 59 (3), 997–1007. doi: 10.1093/jme/tjac006 35139201

[B26] Estevam L. G. T. M. Fonseca Junior A. A. Silvestre B. T. Hemetrio N. S. Almeida L. R. Oliveira M. M.. (2020). Seven years of evaluation of ectoparasites and vector-borne pathogens among ring-tailed coatis in an urban park in southeastern Brazil. Vet. Parasitol. Reg. Stud. Rep. 21, 100442. doi: 10.1016/j.vprsr.2020.100442 32862904

[B27] Falcão de Oliveira E. Oliveira A. G. Arruda C. C. P. Fernandes W. S. Medeiros M. J. (2020). Spatio-temporal modeling of visceral leishmaniasis in Midwest Brazil: An ecological study of 18-years data, (2001-2018). PloS One 15 (10), e0240218. doi: 10.1371/journal.pone.0240218 33007033 PMC7531797

[B28] Figueiredo F. B. Gremião I. D. Pereira S. A. Fedulo L. P. Menezes R. C. Balthazar D. A.. (2008). First report of natural infection of a bush dog (*Speothos venaticus*) with *Leishmania* (*Leishmania*) *chagasi* in Brazil. Trans. R. Soc Trop. Med. Hyg. 102 (2), 200–201. doi: 10.1016/j.trstmh.2007.10.001 18036627

[B29] Furlan M. B. G. (2010). Visceral leishmaniasis epidemic in campo grande, state of mato grosso do sul, Brazil, from 2002 to 2006. Epidemiol. Serv. Saúde. 19 (1), 16–25. doi: 10.5123/S1679-49742010000100003

[B30] Gompper M. E. Decker D. M. (1998). Nasua nasua. Mammn Species. 580, 1–9. doi: 10.2307/3504444

[B31] Graça G. C. Volpini A. C. Romero G. A. Oliveira Neto M. P. Hueb M. Porrozzi R.. (2012). Development and validation of PCR-based assays for diagnosis of American cutaneous leishmaniasis and identification of the parasite species. Mem. Inst. Oswaldo Cruz. 107 (5), 664–674. doi: 10.1590/s0074-02762012000500014 22850958

[B32] Guerra-Silveira F. Abad-Franch F. (2013). Sex bias in infectious disease epidemiology: patterns and processes. PloS One 8 (4), e62390. doi: 10.1371/journal.pone.0062390 23638062 PMC3634762

[B33] Herrera G. Barragán N. Luna N. Martínez D. De Martino F. Medina J.. (2020). An interactive database of *Leishmania* species distribution in the americas. Sci. Data. 7 (1), 110. doi: 10.1038/s41597-020-0451-5 32245983 PMC7125201

[B34] Herrera H. M. Dávila A. M. Norek A. Abreu U. G. Souza S. S. D’Andrea P. S.. (2004). Enzootiology of *Trypanosoma evansi* in pantanal, Brazil. Vet. Parasitol. 125 (3-4), 263–275. doi: 10.1016/j.vetpar.2004.07.013 15482883

[B35] Humberg R. M. Oshiro E. T. Cruz M. do S. Ribolla P. E. Alonso D. P.. (2012). *Leishmania chagasi* in opossums (*Didelphis albiventris*) in an urban area endemic for visceral leishmaniasis, campo grande, mato grosso do sul, Brazil. Am. J. Trop. Med. Hyg. 87 (3), 470–472. doi: 10.4269/ajtmh.2012.11-0534 22802435 PMC3435349

[B36] Jusi M. M. Starke-Buzetti W. A. Oliveira T. M. Tenório M. da S. de O. L.. (2011). Molecular and serological detection of leishmania spp. in captive wild animals from ilha solteira, SP, Brazil. Rev. Bras. Parasitol. Vet. 20 (3), 219–222. doi: 10.1590/s1984-29612011000300008 21961752

[B37] Keesing F. Ostfeld R. S. (2021). Dilution effects in disease ecology. Ecol. Lett. 24 (11), 2490–2505. doi: 10.1111/ele.13875 34482609 PMC9291114

[B38] Lainson R. Braga R. R. Souza A. A. Povoa M. M. Ishikawa E. A. Silveira F. T. (1989). *Leishmania* (*Viannia*) shawi sp.n., a parasite of monkeys, sloths and procyonids in Amazonian Brazil. Ann. Parasitol. Hum. Comp. 64 (3), 200–207. doi: 10.1051/parasite/1989643200 2504099

[B39] Lainson R. Dye C. Shaw J. J. Macdonald D. W. Courtenay O. Souza A. A.. (1990). Amazonian Visceral leishmaniasis–distribution of the vector *Lutzomyia longipalpis* (Lutz & neiva) in relation to the fox *Cerdocyon thous* (linn.) and the efficiency of this reservoir host as a source of infection. Mem. Inst. Oswaldo Cruz. 85 (1), 135–137. doi: 10.1590/s0074-02761990000100027 2215228

[B40] Lima B. S. Dantas-Torres F. de Carvalho M. R. Marinho-Junior J. F. de Almeida E. L. Brito M. E.. (2013). Small mammals as hosts of leishmania spp. in a highly endemic area for zoonotic leishmaniasis in north-Eastern Brazil. Trans. R. Soc Trop. Med. Hyg. 107 (9), 592–597. doi: 10.1093/trstmh/trt062 23868744

[B41] Lockard R. D. Wilson M. E. Rodríguez N. E. (2019). Sex-related differences in immune response and symptomatic manifestations to infection with *Leishmania* species. J. Immunol. Res. 2019, 4103819. doi: 10.1155/2019/4103819 30756088 PMC6348913

[B42] Maia C. Cristóvão J. Pereira A. Kostalova T. Lestinova T. Sumova P.. (2020). Monitoring *Leishmania* infection and exposure to *Phlebotomus perniciosus* using minimal and non-invasive canine samples. Parasitol. Vectors. 13 (1), 119. doi: 10.1186/s13071-020-3993-7 PMC7171869 32312325

[B43] Metzdorf I. P. da Costa Lima M. S. Jr de Fatima Cepa Matos M. de Souza Filho A. F. de Souza Tsujisaki R. A. Franco K. G.. (2017). Molecular characterization of *Leishmania infantum* in domestic cats in a region of Brazil endemic for human and canine visceral leishmaniasis. Acta Trop. 166, 121–125. doi: 10.1016/j.actatropica.2016.11.013 27851895

[B44] Miró G. Montoya A. Mateo M. Alonso A. García S. García A.. (2007). A leishmaniosis surveillance system among stray dogs in the region of Madrid: ten years of serodiagnosis, (1996-2006). Parasitol. Res. 101 (2), 253–257. doi: 10.1007/s00436-007-0497-8 17323100

[B45] Mol J. P. Soave S. A. Turchetti A. P. Pinheiro G. R. Pessanha A. T. Malta M. C.. (2015). Transmissibility of *Leishmania infantum* from maned wolves (*Chrysocyon brachyurus*) and bush dogs (*Speothos venaticus*) to *Lutzomyia longipalpis* . Vet. Parasitol. 212 (3-4), 86–91. doi: 10.1016/j.vetpar.2015.08.024 26342623

[B46] MS-BR (2019) Vigilância em saúde no brasil 2003/2019: da criação da secretaria de vigilância em saúde aos dias atuais. ministério da saúde (BR). Available at: http://www.rets.epsjv.fiocruz.br/biblioteca/vigilancia-em-saude-no-brasil-20032019-da-criacao-dasecretaria-de-vigilancia-em-saude (Accessed January 23, 2022).

[B47] MS-BR (2021) DATASUS: informações de saúde: informações epidemiológicas e morbidade (Ministério da Saúde (BR). Available at: https://datasus.saude.gov.br/epidemiologicas-e-morbidade/ (Accessed January 25, 2022).

[B48] Müller A. Montoya A. Escacena C. de la Cruz M. Junco A. Iriso A.. (2022). *Leishmania infantum* infection serosurveillance in stray dogs inhabiting the Madrid community: 2007–2018. Parasitol. Vectors. 15 (1), 96. doi: 10.1186/s13071-022-05226-6 PMC9281004 35422058

[B49] Nantes W. A. G. Santos F. M. de Macedo G. C. Barreto W. T. G. Gonçalves L. R. Rodrigues M. S.. (2021). Trypanosomatid species in *Didelphis albiventris* from urban forest fragments. Parasitol. Res. 120 (1), 223–231. doi: 10.1007/s00436-020-06921-y 33079269

[B50] Oliva G. Scalone A. Foglia Manzillo V. Gramiccia M. Pagano A. Di Muccio T.. (2006). Incidence and time course of *Leishmania infantum* infections examined by parasitological, serologic, and nested-PCR techniques in a cohort of naive dogs exposed to three consecutive transmission seasons. J. Clin. Microbiol. 44 (4), 1318–1322. doi: 10.1128/JCM.44.4.1318-1322.2006 16597857 PMC1448675

[B51] Oliveira A. G. Galati E. A. Fernandes C. E. Dorval M. E. Brazil R. P. (2012). Ecological aspects of phlebotomines (Diptera: Psychodidae) in endemic area of visceral leishmaniasis, campo grande, state of mato grosso do sul, Brazil. J. Med. Entomol. 49 (1), 43–50. doi: 10.1603/me11082 22308770

[B52] Oliveira F. S. Pirmez C. Pires M. Q. Brazil R. P. Pacheco R. S. (2005). PCR-based diagnosis for detection of *Leishmania* in skin and blood of rodents from an endemic area of cutaneous and visceral leishmaniasis in Brazil. Vet. Parasitol. 129 (3-4), 219–227. doi: 10.1016/j.vetpar.2005.01.005 15845276

[B53] Ostyn B. Gidwani K. Khanal B. Picado A. Chappuis F. Singh S. P.. (2011). Incidence of symptomatic and asymptomatic *Leishmania donovani* infections in high-endemic foci in India and Nepal: A prospective study. PloS Negl. Trop. Dis. 5 (10), e1284. doi: 10.1371/journal.pntd.0001284 21991397 PMC3186756

[B54] PAHO (2020) Leishmaniases: epidemiological report of the americas (Pan American Health Organization). Available at: https://iris.paho.org/handle/10665.2/53090 (Accessed December 15, 2021).

[B55] Paiz L. M. Fornazari F. Menozzi B. D. Oliveira G. C. Coiro C. J. Teixeira C. R.. (2015). Serological evidence of infection by *Leishmania* (*Leishmania*) *infantum* (Synonym: *Leishmania* (*Leishmania*) *chagasi*) in free-ranging wild mammals in a nonendemic region of the state of são paulo, Brazil. Vector Borne Zoonotic Dis. 15 (11), 667–673. doi: 10.1089/vbz.2015.1806 26418884

[B56] Paranhos-Silva M. Oliveira G. G. Reis E. A. de Menezes R. M. Fernandes O. Sherlock I.. (2003). A follow-up of beagle dogs intradermally infected with *Leishmania chagasi* in the presence or absence of sand fly saliva. Vet. Parasitol. 114 (2), 97–111. doi: 10.1016/s0304-4017(03)00132-8 12781472 PMC7126804

[B57] Porfirio G. E. O. Santos F. M. de Macedo G. C. Barreto W. T. G. Campos J. B. V. Meyers A. C.. (2018). Maintenance of *Trypanosoma cruzi*, t. evansi and leishmania spp. by domestic dogs and wild mammals in a rural settlement in Brazil-Bolivian border. Int. J. Parasitol: Parasites Wildl. 7 (3), 398–404. doi: 10.1016/j.ijppaw.2018.10.004 30370220 PMC6199764

[B58] Quinnell R. J. Courtenay O. (2009). Transmission, reservoir hosts and control of zoonotic visceral leishmaniasis. Parasitol 136 (14), 1915–1934. doi: 10.1017/S0031182009991156 19835643

[B59] R Development Core Team (2015) R: a language and environment for statistical computing. Available at: http://www.R-project.org (Accessed September 22, 2021).

[B60] Reis F. C. Minuzzi-Souza T. T. C. Neiva M. Timbó R. V. de Morais I. O. B. de Lima T. M.. (2020). Trypanosomatid infections in captive wild mammals and potential vectors at the Brasilia zoo, federal district, Brazil. Vet. Med. Sci. 6 (2), 248–256. doi: 10.1002/vms3.216 31743623 PMC7196675

[B61] Risueño J. Ortuño M. Pérez-Cutillas P. Goyena E. Maia C. Cortes S.. (2018). Epidemiological and genetic studies suggest a common *Leishmania infantum* transmission cycle in wildlife, dogs and humans associated to vector abundance in southeast Spain. Vet. Parasitol. 259, 61–67. doi: 10.1016/j.vetpar.2018.05.012 30056986

[B62] Rodrigues E. S. Santos G. Q. da Silva M. V. Barros J. H. S. Bernardo A. R. Diniz R. L.. (2022). Chagas immunochromatographic rapid test in the serological diagnosis of *Trypanosoma cruzi* infection in wild and domestic canids. Front. Cell Infect. Microbiol. 12. doi: 10.3389/fcimb.2022.835383 PMC8902141 35273924

[B63] Roque A. L. Jansen A. M. (2014). Wild and synanthropic reservoirs of *Leishmania* species in the americas. Int. J. Parasitol: Parasites Wildl. 3 (3), 251–262. doi: 10.1016/j.ijppaw.2014.08.004 25426421 PMC4241529

[B64] Salomón O. D. Feliciangeli M. D. Quintana M. G. Afonso M. M. S. Rangel E. F. (2015). *Lutzomyia longipalpis* urbanisation and control. Mem. Inst. Oswaldo Cruz. 110 (7), 831–846. doi: 10.1590/0074-02760150207 26517497 PMC4660613

[B65] Sambrook J. Russell D. W. (2001). Molecular cloning – a laboratory manual (New York: Cold Spring Harbor Laboratory Press).

[B66] Schubach A. Haddad F. Oliveira-Neto M. P. Degrave W. Pirmez C. Grimaldi G. Jr. (1998). Detection of *Leishmania* DNA by polymerase chain reaction in scars of treated human patients. J. Infect. Dis. 178 (3), 911–914. doi: 10.1086/515355 9728572

[B67] SES/MS (2022) Boletim epidemiológico da leishmaniose visceral humana de 2021 (Secretaria do Estado de Saúde de Mato Grosso do Sul). Available at: https://www.vs.saude.ms.gov.br/Geral/vigilancia-saude/vigilancia-epidemiologica/boletim-epidemiologico/leishmaniose/ (Accessed January 15, 2021).

[B68] Silva E. A. Andreotti R. Dias E. S. Barros J. C. Brazuna J. C. (2008). Detection of *Leishmania* DNA in phlebotomines captured in campo grande, mato grosso do sul, Brazil. Exp. Parasitol. 119 (3), 343–348. doi: 10.1016/j.exppara.2008.03.011 18456262

[B69] Silva E. S. Pirmez C. Gontijo C. M. Fernandes O. Brazil R. P. (2000). Visceral leishmaniasis in the crab-eating fox (*Cerdocyon thous*) in south-east Brazil. Vet. Rec. 147 (15), 421–422. doi: 10.1136/vr.147.15.421 11072988

[B70] Souza N. P. Almeida A. do B. Freitas T. P. Paz R. C. Dutra V.. (2010). *Leishmania* (*Leishmania*) *infantum chagasi* in wild canids kept in captivity in the state of mato grosso. Rev. Soc Bras. Med. Trop. 43 (3), 333–335. doi: 10.1590/s0037-86822010000300024 20563507

[B71] Talmi-Frank D. Nasereddin A. Schnur L. F. Schönian G. Töz S. O. Jaffe C. L.. (2010). Detection and identification of old world *Leishmania* by high resolution melt analysis. PloS Negl. Trop. Dis. 4 (1), e581. doi: 10.1371/journal.pntd.0000581 20069036 PMC2797090

[B72] Vásquez Trujillo A. González Reina A. E. Góngora Orjuela A. Prieto Suárez E. Palomares J. E. Buitrago Alvarez L. S. (2013). Seasonal variation and natural infection of *Lutzomyia antunesi* (Diptera: Psychodidae: Phlebotominae), an endemic species in the orinoquia region of Colombia. Mem. Inst. Oswaldo Cruz. 108 (4), 463–469. doi: 10.1590/S0074-0276108042013011 23828011 PMC3970617

[B73] Vieira T. M. Silva S. O. Lima L. Sabino-Santos G. Duarte E. R. Lima S. M.. (2022). *Leishmania* diversity in bats from an endemic area for visceral and cutaneous leishmaniasis in southeastern Brazil. Acta Trop. 228, 106327. doi: 10.1016/j.actatropica.2022.106327 35085511

[B74] Voltarelli E. M. Arraes S. M. A. A. Perles T. F. Lonardoni M. V. C. Teodoro U. Silveira T. G. V. (2009). Serological survey for leishmania spp. infection in wild animals from the municipality of maringá, paraná state, Brazil. J. Venom. Anim. Toxins incl. Trop. Dis. 15 (4), 732–744. doi: 10.1590/S1678-91992009000400011

