# Corrigendum: *Leishmania infantum* infecting the carnivore *Nasua nasua* from urban forest fragments in an endemic area of visceral leishmaniasis in Brazilian Midwest

**DOI:** 10.3389/fcimb.2023.1155813

**Published:** 2023-03-03

**Authors:** Gabriel Carvalho de Macedo, Wanessa Teixeira Gomes Barreto, Carina Elisei de Oliveira, Filipe Martins Santos, Grasiela Edith de Oliveira Porfírio, Samanta Cristina das Chagas Xavier, Fernanda Moreira Alves, Alanderson Rodrigues da Silva, Gisele Braziliano de Andrade, Andreza Castro Rucco, William Oliveira de Assis, Ana Maria Jansen, André Luiz Rodrigues Roque, Heitor Miraglia Herrera

**Affiliations:** ^1^ Post-Graduate Program in Environmental Sciences and Agricultural Sustainability, Dom Bosco Catholic University, Campo Grande, Brazil; ^2^ Post-Graduate Program in Biotechnology, Dom Bosco Catholic University, Campo Grande, Brazil; ^3^ Laboratory of Trypanosomatid Biology, Oswaldo Cruz Institute, Oswaldo Cruz Foundation, Rio de Janeiro, Brazil; ^4^ Post-Graduate Program in Parasite Biology, Oswaldo Cruz Institute, Oswaldo Cruz Foundation, Rio de Janeiro, Brazil

**Keywords:** longitudinal study, South American coati, *Leishmania infantum*, urban fauna, visceral leishmaniasis

## Error in Figure

In the published article, there was an error in **Figure 1** as published. **Figure 1B** and **Figure 1C** were not corresponding to the correct areas. The corrected **Figure 1** and its caption appear below. The authors apologize for this error and state that this does not change the scientific conclusions of the article in any way. The original article has been updated.

**Figure 1:**
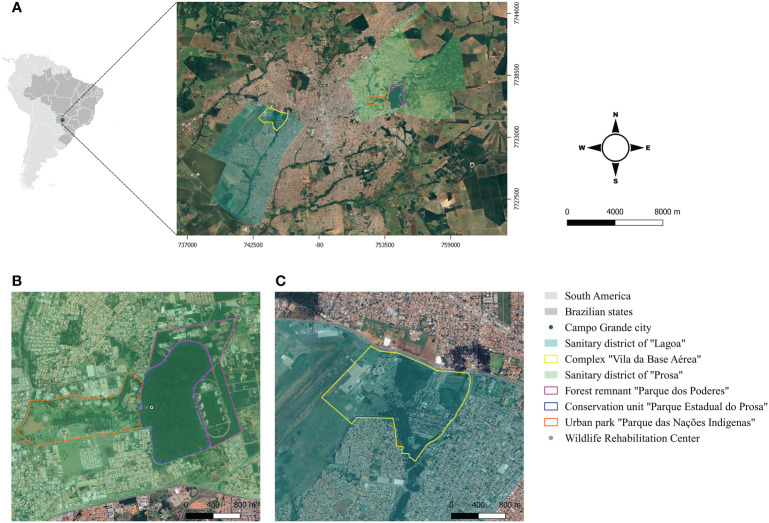
**(A)** Geographical location of the study areas in Campo Grande, Mato Grosso do Sul, Midwest Brazil; **(B)** Conservation unit “Parque Estadual do Prosa” and its adjacent areas “Parque dos Poderes” and “Parque das Nações Indígenas”; **(C)** Complex “Vila da Base Aeríea”.

